# Prognostic Biomarkers of Sarcoidosis: A Comparative Study of Serum Chitotriosidase, ACE, Lysozyme, and KL-6

**DOI:** 10.1155/2019/8565423

**Published:** 2019-03-03

**Authors:** Laura Bergantini, Francesco Bianchi, Paolo Cameli, Maria Antonietta Mazzei, Annalisa Fui, Piersante Sestini, Paola Rottoli, Elena Bargagli

**Affiliations:** ^1^Department of Clinical Medicine and Immunological Sciences, Respiratory Disease and Lung Transplant Unit, Respiratory Diseases and Transplant Unit, Siena University, Siena, Italy; ^2^Department of Clinical Medicine and Immunological Sciences, Radiology Unit, Siena University, Siena, Italy

## Abstract

**Purpose:**

Sarcoidosis is a systemic granulomatous disease with unknown etiology. Many clinical presentations have been reported, and acute disease needs to be distinguished from subacute and chronic disease. The unpredictable clinical course of the disease prompted us to evaluate the clinical utility of biomarker serum detection in sarcoidosis follow-up.

**Methods:**

Serum concentrations of chitotriosidase, ACE, KL-6, and lysozyme were analyzed by different methods in a population of 74 sarcoidosis patients (46 on steroid therapy at sampling) regularly monitored at Siena Sarcoidosis Regional Referral Centre and in a group of controls with the aim of comparing their contribution to clinical management of sarcoidosis patients.

**Results:**

KL-6 concentrations were significantly elevated in sarcoidosis patients with lung fibrosis and were significantly correlated with DLco and CPI score, while chitotriosidase was significantly higher in patients with extrapulmonary localizations. With a cut-off value of 303.5 IU/ml, KL-6 showed the best sensitivity (78%), while chitotriosidase reported the best specificity (85%) among the biomarkers.

**Conclusions:**

KL-6 is a reliable biomarker of fibrotic lung involvement in sarcoidosis patients. Among biomarkers, KL-6 showed the best sensitivity and serum chitotriosidase the best specificity, even in patients on chronic steroid therapy, and seemed to correlate with extrapulmonary localizations.

## 1. Introduction

Sarcoidosis is a systemic granulomatous disease associated with T lymphocyte and macrophage activation and migration into affected organs. The interaction between antigens and APC polarizes T lymphocytes to T helper 1 phenotype (Th1), leading to the formation of sarcoid granulomas consisting of T cells, macrophages, and epithelioid and giant cells [1, 2]. The course of sarcoidosis is unpredictable: remission occurs in most cases, while persistent granuloma inflammation may lead to fibrotic lung disease [3–6]. Specific biomarkers with good sensitivity and specificity are therefore needed to predict clinical outcome and guide clinical decisions.

Human chitotriosidase is a biomarker secreted by activated macrophages and neutrophils. Although its physiological role is not clear, it may play a role in the hydrolysis and degradation of chitin and chitin-like substrates [7]. Increased concentrations of chitotriosidase have been reported in serum and BAL of patients with active sarcoidosis [8]. Chitotriosidase shows a correlation with radiological stages. It may predict clinical course, steroid responsiveness, and potential relapses of the disease [9]. However, it is not clear whether chitotriosidase is reliable in the management of sarcoidosis patients with extrapulmonary organ involvement.

Angiotensin-converting enzyme (ACE) is an acid glycoprotein that converts angiotensin I into angiotensin II. It is produced by lung endothelial cells, mainly activated alveolar macrophages [10]. It is elevated in serum and BAL of sarcoidosis patients and its concentrations correlate with radiological stages [11–13]. However, its utility as a diagnostic and prognostic tool is limited by its low sensitivity and specificity. ACE may be elevated in certain granulomatous disorders (including berylliosis and silicosis), hyperthyroidism, diabetes, and other diseases [14, 15].

Lysozyme is produced by monocyte-macrophage system and epithelioid cells involved in granuloma formation can release this enzyme. Sarcoidosis patients show increased concentration of lysozyme at onset. Lysozyme is regarded more as a prognostic indicator rather than a diagnostic tool. It has a low specificity being elevated in lung diseases such as tuberculosis and pneumoconiosis [16, 17].

Krebs von den Lungen-6 (KL-6) is a human high-molecular weight MUC1 mucin protein derived from AEC. Its serum concentrations are elevated in several ILDs, including idiopathic pulmonary fibrosis and sarcoidosis [18–20]. In sarcoidosis, KL-6 correlates with ACE activity: it is mainly increased in stage 2 and 3 patients with scintigraphic evidence of positive pulmonary accumulation [21]. High levels of serum KL-6 in sarcoidosis reflect production of KL-6 derived from damaged or regenerating type 2 pneumocytes [22].

In this study, we compared serum levels of different biomarkers in a cohort of patients with chronic sarcoidosis looked for correlations with specific phenotypes, clinical presentation, and localizations.

## 2. Materials and Methods

### 2.1. Study Population

We retrospectively enrolled 74 sarcoidosis patients (27 males (36.5%), with a mean age of 44.6 ± 7.7 years) regularly monitored at Siena Regional Referral Centre for Sarcoidosis and ILDs. All patients were diagnosed according to international ATS/ERS/WASOG criteria. All had been in follow-up at our center for more than 2 years since diagnosis and showed a persistent chronic disease. The exclusion criteria included patients with Lofgren syndrome, acute disease onset, spontaneous resolution, or a follow-up of less than 2 years.

Serum sampling was performed at the enrollment: in the same day, pulmonary function test (PFT) and chest X-ray, with radiological staging according to Scadding criteria [23], were performed. The radiological classification was linked to sample detection in a standard manner according to widely accepted criteria: stage 0, normal; stage 1, bilateral hilar adenopathy without parenchymal involvement; stage 2, bilateral adenopathy and parenchymal infiltration; stage 3, parenchymal infiltration; and stage 4, pulmonary fibrosis associated with sarcoidosis. All patients underwent high-resolution CT scan of the chest (HRCT) to check for pulmonary fibrosis. All subjects gave their informed consent to the study, approved by the Local Ethic Committee. In order to find a cut-off value of serum KL-6 in our population, we collected serum samples from 25 healthy volunteers (6 males, mean age 48 ± 21 years), with no history of respiratory diseases and not taking any drugs

### 2.2. Pulmonary Function Tests

The following lung function measurements were conducted according to ATS/ERS standard parameters [24–26], using a Jaeger Body Plethysmograph with corrections for temperature and barometric pressure: forced expiratory volume in the first second (FEV1), forced vital capacity (FVC), and diffuse lung carbon monoxide (DLCO). All parameters were expressed as percentages of predicted values [27].

### 2.3. Clinical Phenotyping

Patients were classified on the basis of the radiological lung involvement and sarcoidosis localizations, according to recent Genotype-Phenotype Relationship in Sarcoidosis document (GenPhenReSa) [28], as well as composite physiologic index (CPI) score. We calculated CPI in every patient according to the following formula, as previously described [29]: 91 − (0.65 × percent predicted DLCO) − (0.53 × percent predicted FVC) + (0.34 × percent predicted FEV1).

Extrapulmonary localizations of disease were assessed with organ-specific diagnostic pathways: involvements of the liver, spleen, bone, bone marrow, extrathoracic lymph nodes, and skin were all biopsy proven, while cardiac localization was assessed with MR imaging.

### 2.4. Chitotriosidase Assay

Chitotriosidase activity was determined by a fluorimetric method using 22 *μ*M 4-methylumbelliferyl *β*-D-N,N′,N^″^-triacetylchitotriosidase (Sigma Chemical Co.) in citrate-phosphate buffer, pH 5.2; 100 *μ*l substrate was incubated for 1 h at 37°C and the reaction was stopped with 1.4 ml 0.1 M glycine-NaOH buffer, pH 10.8. Fluorescence was read at 450 nm with a PerkinElmer Victor X4 fluorimeter (excitation wavelength 365 nm). Serum activity of chitotriosidase was expressed in nmol/ml/h. Normality values were calculated according to our previous studies [9].

### 2.5. ACE Assay

ACE activity was measured using a colorimetric method (FAR srl, Verona, Italy), for determination of ACE activity in serum. The normal range of ACE concentrations was 30-80 IU/l [30].

### 2.6. Lysozyme Assay

Lysozyme activity was measured using a colorimetric method (FAR kit, FAR srl, Verona, Italy). The reference value for serum lysozyme was 2.5-8 mg/l [17].

### 2.7. Krebs von den Lungen-6 Assay

Serum KL-6 was measured by NANOPIA® KL-6 reagents assay (Sekisui Diagnostics, UK). The principle of the assay is agglutination of sialylated carbohydrate antigen in samples with KL-6 monoclonal antibody through the antigen-antibody reaction. The change in absorbance is measured to determine serum KL-6 levels. KL-6 concentrations are expressed in IU/ml.

### 2.8. Statistical Analysis

The data was expressed as mean ± standard deviation (M ± SD). Comparisons between groups were performed by Mann-Whitney test and Kruskal-Wallis test with significance set at *p* < 0.05. The Spearman test was used to look for correlations between variables. Statistical analysis and ROC curves were performed using Statistica v 7.0 software; graphic representations of data were conducted using GraphPad Prism 4.0 software.

## 3. Results

### 3.1. Clinical, Radiological, and Functional Parameters

Demographic features, pulmonary function test values, and Scadding radiological stages of population are reported in Table 1. As expected [31], onset mainly occurred in the 5th decade (44.6 ± 7.7 years), prevalent in never-smoker females. Concerning medical history, 46 patients (62%, 17 males) were on steroid therapy at the time of serum sampling, with an average dose of 7.07 ± 9.74 mg of prednisone. Of these, all but one were taking oral steroids for more than one year. 13 patients (3 males) were taking immunosuppressive drugs in combination with oral steroids (12 with methotrexate, 1 with azathioprine). Among steroid-free subgroup (28 patients, 10 males), only two patients had never taken specific therapy for sarcoidosis.

In our population, PFT parameters were in the normal range with no significant alteration of lung volumes: 63 patients managed to perform an acceptable maneuver for DLCO that was mildly impaired, on average.

### 3.2. Comparison of Biomarkers

Serum KL-6 concentration was calculated in all patients and healthy volunteers; ACE and lysozyme were detected in serum of all patients. Six patients with chitotriosidase activity <10 nmol/ml/h were suspected to have CHIT1 polymorphism and were excluded from the analysis.

Sarcoidosis patients reported significantly higher KL-6 levels than healthy controls (573 ± 480 IU/ml vs. 267.7 ± 147.7 IU/ml, *U* = 344, *p* < 0.0001). ROC curve analysis revealed an area under the curve (AUC) of 0.788, 78% sensitivity and 73% specificity, with a cut-off of 303.5 IU/ml (*p* < 0.0001). Table 1 shows the mean concentrations of serum biomarkers in our population: the prevalence of elevated KL-6, chitotriosidase, ACE, and lysozyme was 78%, 58.1%, 36.5%, and 12.1%, respectively.

No significant differences of biomarkers' levels were found between patients treated with steroids and those treated with steroids and immunosuppressive therapy.

**Figure 1:**
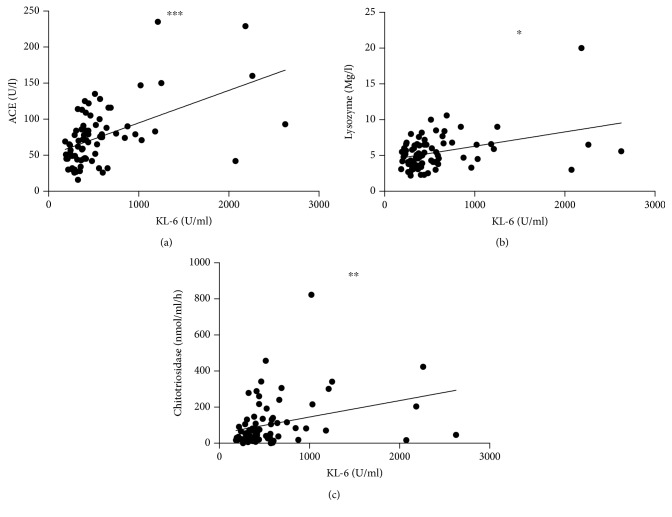
KL-6 correlations with ACE, chitotriosidase, and lysozyme. ^∗∗∗^*r* = 0.5154, *p* < 0.0001; ^∗^*r* = 0.3238, *p* = 0.0049; ^∗∗^*r* = 0.3750, *p* = 0.001.

KL-6 was significantly correlated with ACE (*r* = 0.5; *p* < 0.0001), lysozyme (*r* = 0.35; *p* = 0.001), and chitotriosidase activity (*r* = 0.32; *p* = 0.004) (Figure 1).

**Figure 2:**
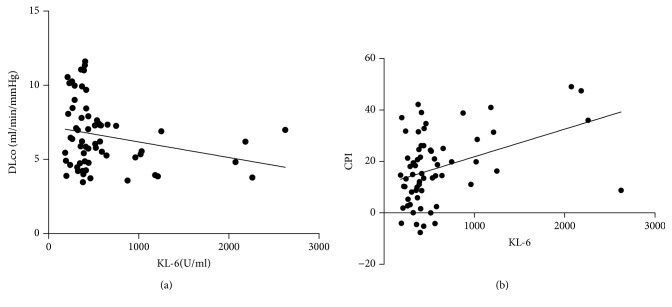
(a) Correlation of KL-6 and DLCO percentages. *R* = -0.34, *p* = 0.0006. (b) Correlation between CPI score and KL-6 levels. CPI: composite physiologic index. *r* = 0.4, *p* < 0.0001.

KL-6 was significantly correlated with DLCO percentages (*r* = -0.34; *p* = 0.006) (Figure 2(a)), but not with FVC, FEV1, and TLC. CPI score (13.6 ± 17.4) showed a significant direct correlation with KL-6 levels (*r* = 0.4; *p* = 0.001) (Figure 2(b)).

### 3.3. Organ Involvement and Phenotypes

**Figure 3:**
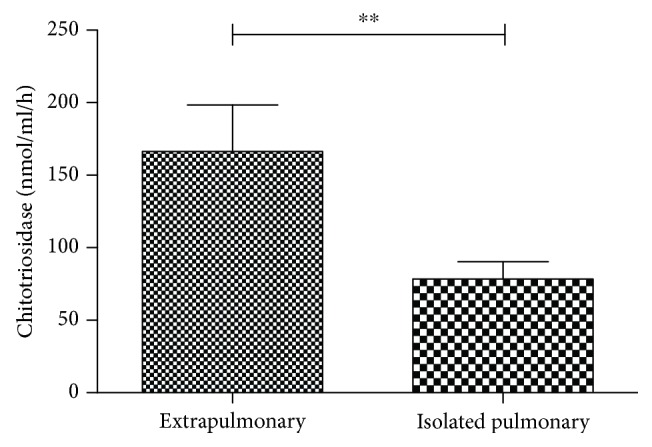
Comparison of chitotriosidase activity between sarcoidosis patients with and without extrapulmonary localizations. ^∗∗^*p* = 0.01.

The percentage of sarcoidosis patients showing extrapulmonary involvement was 43.2%. The most common extrapulmonary localizations were the skin (16 patients, 21.6%), spleen (11 patients, 14.8%), and liver (5 patients, 6.7%). Less common localizations were the eyes (3 patients, 4% of cases) and extrathoracic lymph nodes (3 patients, 4%); CNS (2 patients 2.7% of cases) and cardiac, bone, or bone marrow involvement (1.3% of cases). Sarcoidosis patients with extrapulmonary involvement had significantly higher chitotriosidase activity than those with limited pulmonary disease (146.6 ± 175.9 vs.72.7 ± 80.1 nmol/h/ml; *p* = 0.01) (Figure 3). No other significant differences were observed in relation with organ localizations.

In relation to GenPhenReSa phenotypes, patients with abdominal involvement (*n* = 9) showed significantly higher chitotriosidase activity than those with ocular-cardiac-cutaneous-central nervous system (OCCC) (*n* = 7), musculoskeletal-cutaneous (*n* = 11), and isolated pulmonary involvement (*n* = 34) (221 ± 272 vs.126 ± 162 vs.162 ± 98 vs.86 ± 81, respectively; *p* < 0.05). No statistically significant differences were found between KL-6, ACE, and lysozyme levels in these subgroups.

**Figure 4:**
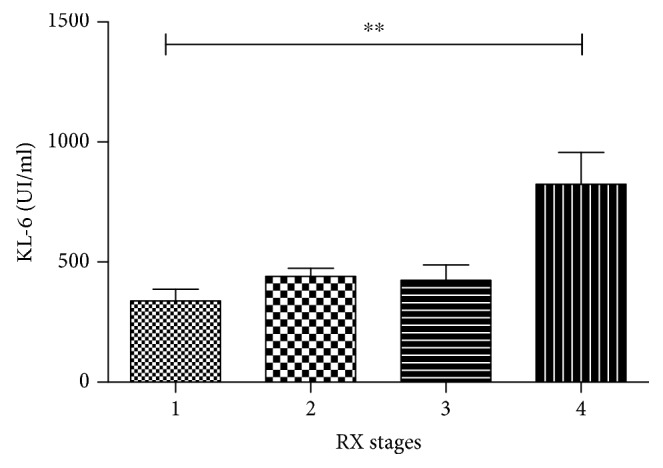
Comparison of KL-6 concentration among RX stages in sarcoidosis population. ^∗∗^*p* = 0.01.

According to radiological score, patients with fibrotic sarcoidosis (stage 4) showed the highest concentrations of serum KL-6 compared to the other radiological stages of disease (*p* = 0.01) (Figure 4).

## 4. Discussion

Many biomarkers have been proposed for sarcoidosis, but none has shown satisfactory prognostic value for identifying relapse under therapy or vital organ localization. In this study, we compared different sarcoidosis biomarkers. We selected chronic disease patients in treatment for more than 1 year with clinical and demographic data compatible with the general features of sarcoidosis reported in the literature [1, 2]. We included KL-6 in the list of biomarkers, although this protein has been studied in IPF and little data are available on its role in sarcoidosis. Our interest in KL-6 was related to its reported correlation with severe fibrotic lung involvement and poor survival [32]. Chitotriosidase, ACE, and lysozyme showed a lower prevalence in our population than previously observed, probably because most patients were on long-term systemic treatment with steroids and did not show signs of active disease at the time of serum sampling. On the contrary, KL-6 showed a good sensitivity and specificity in our population, with a cut-off value of 303.5 IU/ml. To our knowledge, this is the first study to investigate a specific cut-off value of KL-6 in chronic sarcoidosis versus a group of healthy controls; however, this was not the main aim of the study and the data needs to be validated in a wider cohort of patients. Our findings confirmed the predictive value of KL-6 for fibrotic lung involvement and functional impairment in ILD sarcoidosis patients already reported by Miyoshi et al. [20]; however, our study is the first one that shows a significant correlation between a serum biomarker, KL-6, and pulmonary fibrotic lung involvement in sarcoidosis patients, quantified by CPI index. Moreover, this finding is further confirmed by a significant correlation with DLCO percentages, sustaining the potential utility of KL-6 measurements to evaluate sarcoidosis severity in the clinical management of these patients. These data were consistent regardless therapy status, suggesting a chronic macrophage activation unresponsive to steroid therapy, in particular in those patients with fibrotic lung disease. No significant differences of KL-6 expression were found among clinical phenotypes and/or extrapulmonary localizations, indicating that KL-6 can be considered reliable only for pulmonary involvement of disease. Positive correlations were detected among sarcoidosis biomarkers, including ACE, lysozyme, chitotriosidase, and KL-6 that can therefore be included in the list of prognostic indicators.

On the other hand, chitotriosidase proved more sensitive than ACE, lysozyme, and KL-6 even in patients under systemic treatment; it also seems to be the only biomarker that can reflect extrathoracic involvement, according to the recent GenPhenReSa clinical phenotype classification. In particular, its expression is more pronounced in patients with abdominal organ localizations (the liver and spleen, in our population), probably due to an overexpression by activated resident macrophages; this data suggests that chitotriosidase may be a reliable marker of activation of reticuloendothelial system in sarcoidosis, as previously reported in patients with lysosomal storage disease [33]. No studies are available on chitotriosidase prognostic value in splenic and hepatic sarcoidosis localizations and this is a relevant novelty of this study. Unlike KL-6, chitotriosidase activity seems not to be correlated with the severity of sarcoid pulmonary involvement or functional impairment, suggesting that different patterns of macrophage activation may be present in respiratory system of sarcoidosis patients and influence biomarkers' expression [34].

In conclusion, patients with chronic sarcoidosis, severe extrapulmonary involvement, or stage 4 fibrotic impairment represent an important target requiring the identification of reliable prognostic biomarkers to predict disease relapse and response to systemic treatment.

Based on these preliminary results, high levels of chitotriosidase and KL-6 (and not ACE or lsysozyme) were observed in chronic sarcoidosis patients, regardless therapy. Chitotriosidase activity was significantly higher in patients with a multiple organ involvement, showing a promising reliability for evaluation multisistemic sarcoidosis. On the contrary, KL-6 levels correlated with fibrotic lung involvement, CPI score, and reduced DLCO percentages suggest a potential role as a marker of severity of pulmonary sarcoidosis. Interestingly, these findings were reported in a population of chronic multiorgan sarcoidosis patients, including those on therapy, expanding the reliability of chitotriosidase and KL-6 also in this setting.

## Figures and Tables

**Table 1:** Demographic features, pulmonary function test values, radiological assessment, and biomarker assessment in sarcoidosis population.

No.	74
Male (%)	27 (36)
Age (years)	44.6 ± 7.7
Smoking status	
Former (%)	21 (28)
Current (%)	6 (8)
Never (%)	46 (62)
Radiological stages	
0	0
1	7
2	33
3	12
4	22
Pulmonary function tests	
FEV1 %	94.4 ± 22.06
FVC %	104.3 ± 20.2
TLC %	107.7 ± 23.2
DLCO %^∗^	77.4 ± 17.6
Laboratory markers	
ACE (U/l)	76.1 ± 41.9
Lysozyme (mg/l)	5.4 ± 2.5
Chitotriosidase (nmol/ml/h)	105.6 ± 135.8
KL-6 (U/ml)	573 ± 480.7

Data are expressed as media ± SD. ^∗^Parameter available only in 63 patients.

## Data Availability

The datasets generated during and/or analyzed during the current study are available from the corresponding author on reasonable request. Data are available from Laura Bergantini (bergantini@student.unisi.it) and Elena Bargagli (bargagli2@gmail.com) for researchers who meet the criteria for access to confidential data.
